# Supplementary material for: A glyoxal sensing Pseudomonas aeruginosa transcription factor enables lung infection
Source: bioRxiv. 2026 Sep 21:2026.09.20.752901. Preprint. [Version 1] doi: 10.64898/2026.09.20.752901 (PMC13760580; doi:10.64898/2026.09.20.752901)
Supplement: Supplementary file 1 [file media-1.pdf]

Figure S1

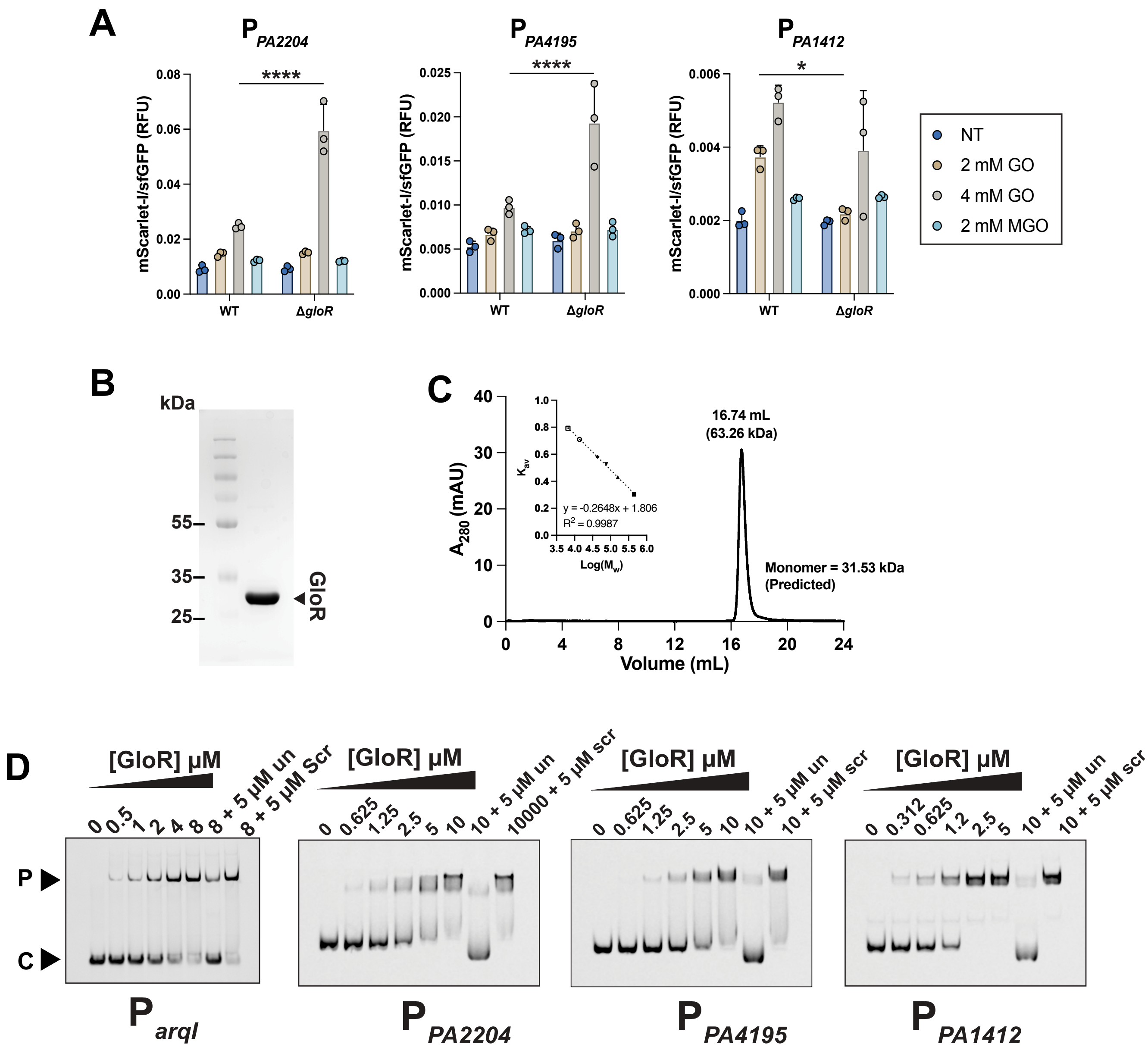

A
P PA2204
P PA4195
P PA1412
mScarlet-I/sfGFP (RFU)
****
****
*
WT
ΔgloR
NT
2 mM GO
4 mM GO
2 mM MGO
B
kDa
55
35
25
GloR
C
16.74 mL
(63.26 kDa)
Monomer = 31.53 kDa
(Predicted)
A280 (mAU)
Volume (mL)
Kav
y = -0.2648x + 1.806
R2 = 0.9987
Log(Mw)
D
[GloR] μM
0 0.5 1 2 4 8 8 + 5 μM un 8 + 5 μM Scr
0 0.625 1.25 2.5 5 10 10 + 5 μM un 10000 + 5 μM scr
0 0.625 1.25 2.5 5 10 10 + 5 μM un 10 + 5 μM scr
0 0.312 0.625 1.2 2.5 5 10 + 5 μM un 10 + 5 μM scr
P
C
P arqI
P PA2204
P PA4195
P PA1412

Figure S2

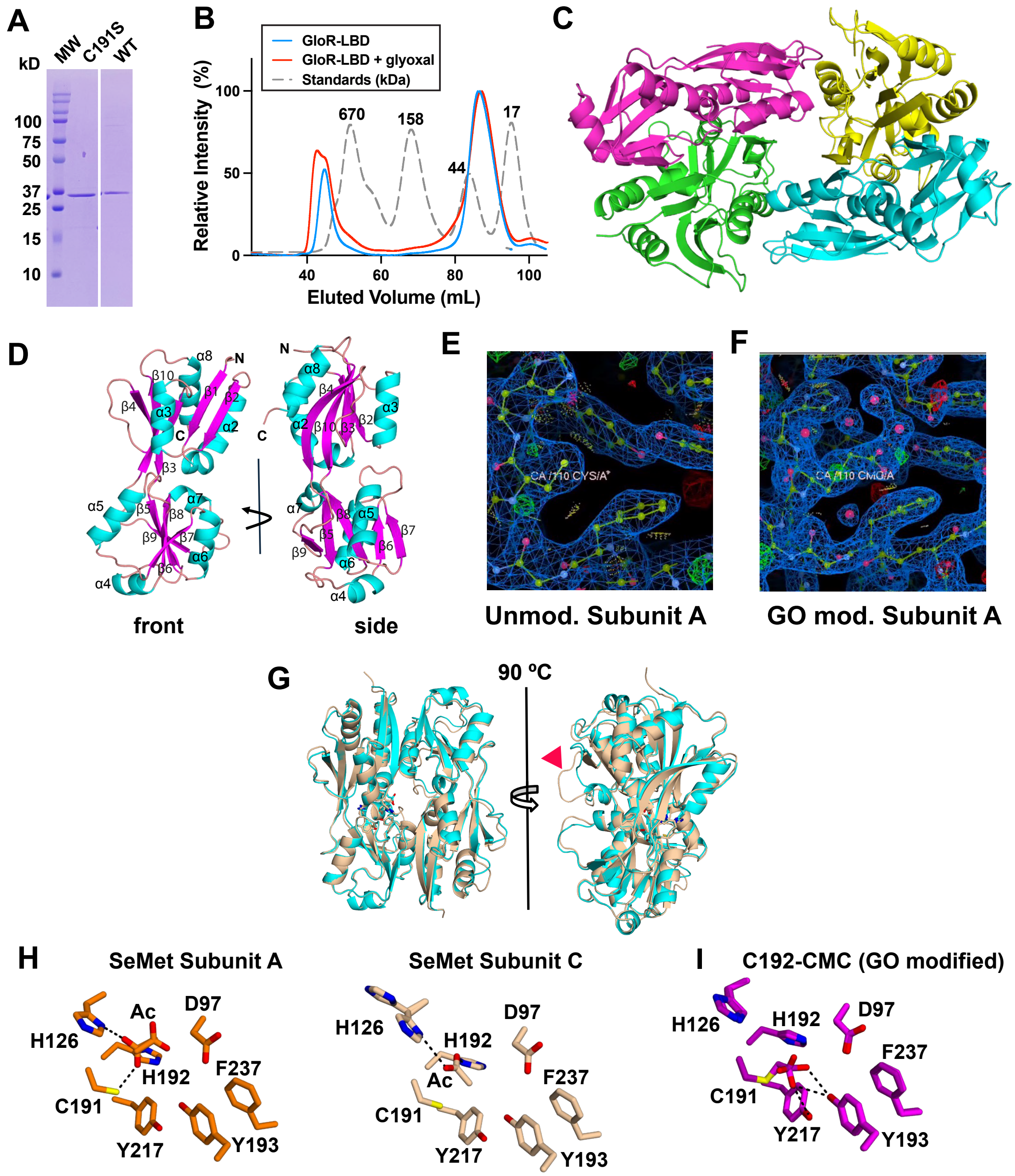
A
kD
MW
C191S
WT
100
75
50
37
25
15
10
B
GloR-LBD
GloR-LBD + glyoxal
Standards (kDa)
Relative Intensity (%)
Eluted Volume (mL)
670
158
44
17
C
D
front
side
E
Unmod. Subunit A
F
GO mod. Subunit A
G
90 ºC
H
SeMet Subunit A
SeMet Subunit C
H126
Ac
D97
H192
F237
C191
Y217
Y193
I
C192-CMC (GO modified)

Figure S3

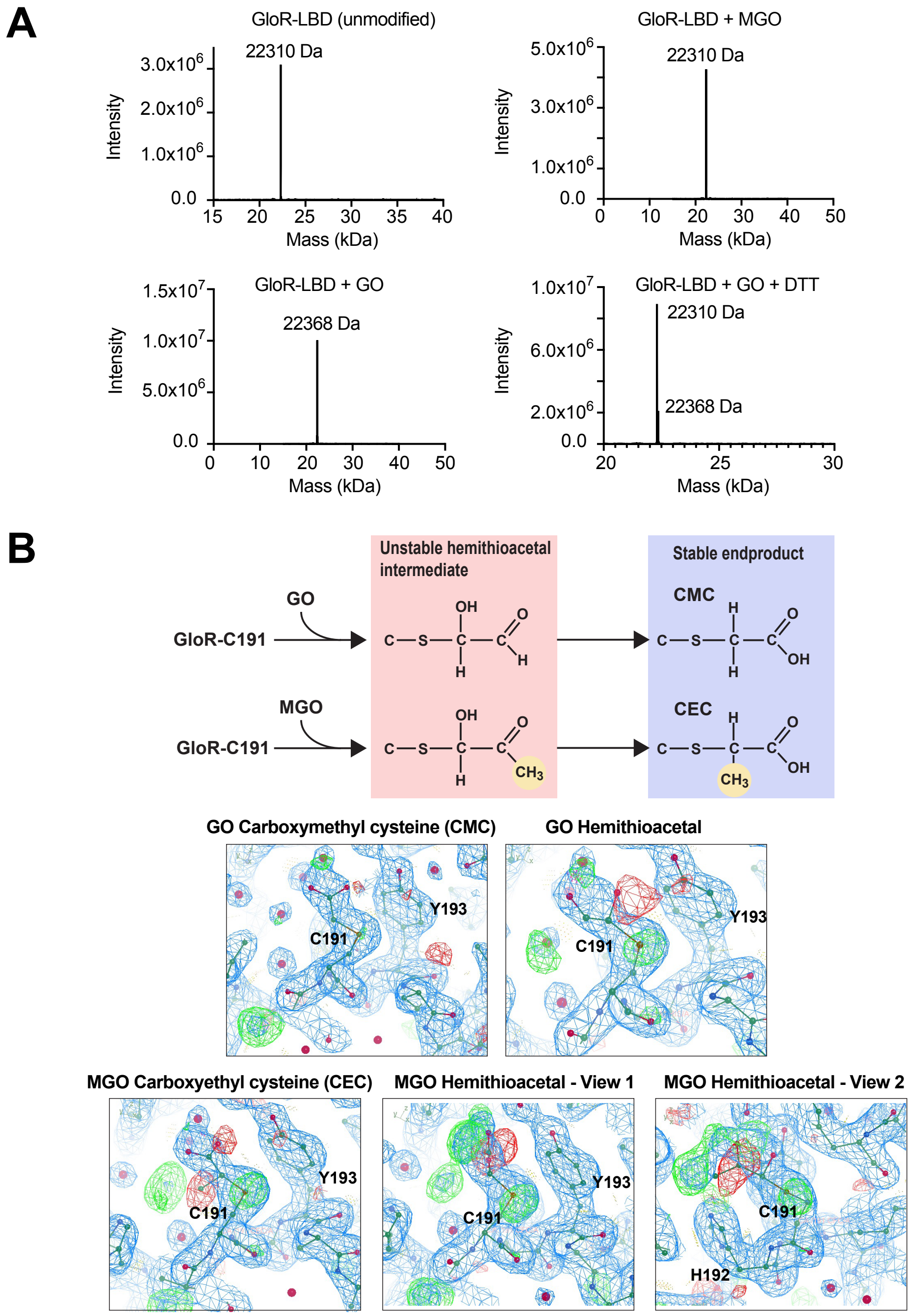
A
GloR-LBD (unmodified)
22310 Da
Intensity
Mass (kDa)
GloR-LBD + MGO
22310 Da
GloR-LBD + GO
22368 Da
GloR-LBD + GO + DTT
22310 Da
22368 Da
B
Unstable hemithioacetal intermediate
Stable endproduct
GO
MGO
GloR-C191
CMC
CEC
GO Carboxymethyl cysteine (CMC)
GO Hemithioacetal
MGO Carboxyethyl cysteine (CEC)
MGO Hemithioacetal - View 1
MGO Hemithioacetal - View 2
C191
Y193
H192

Figure S4

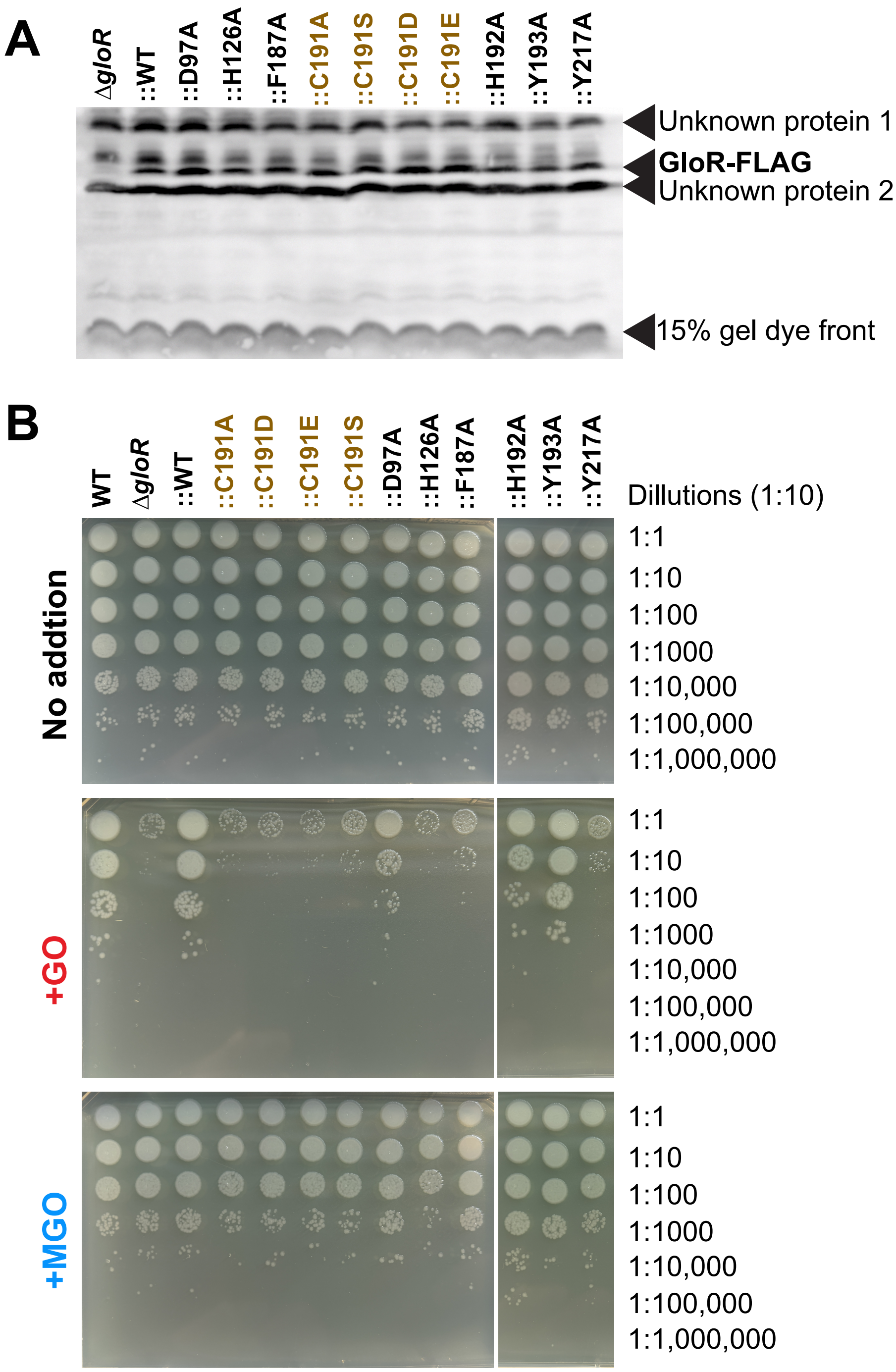
A
ΔgloR
::WT
::D97A
::H126A
::F187A
::C191A
::C191S
::C191D
::C191E
::H192A
::Y193A
::Y217A
Unknown protein 1
GloR-FLAG
Unknown protein 2
15% gel dye front
B
WT
ΔgloR
::WT
::C191A
::C191D
::C191E
::C191S
::D97A
::H126A
::F187A
::H192A
::Y193A
::Y217A
Dillutions (1:10)
No addtion
1:1
1:10
1:100
1:1000
1:10,000
1:100,000
1:1,000,000
+GO
1:1
1:10
1:100
1:1000
1:10,000
1:100,000
1:1,000,000
+MGO
1:1
1:10
1:100
1:1000
1:10,000
1:100,000
1:1,000,000

Figure S5

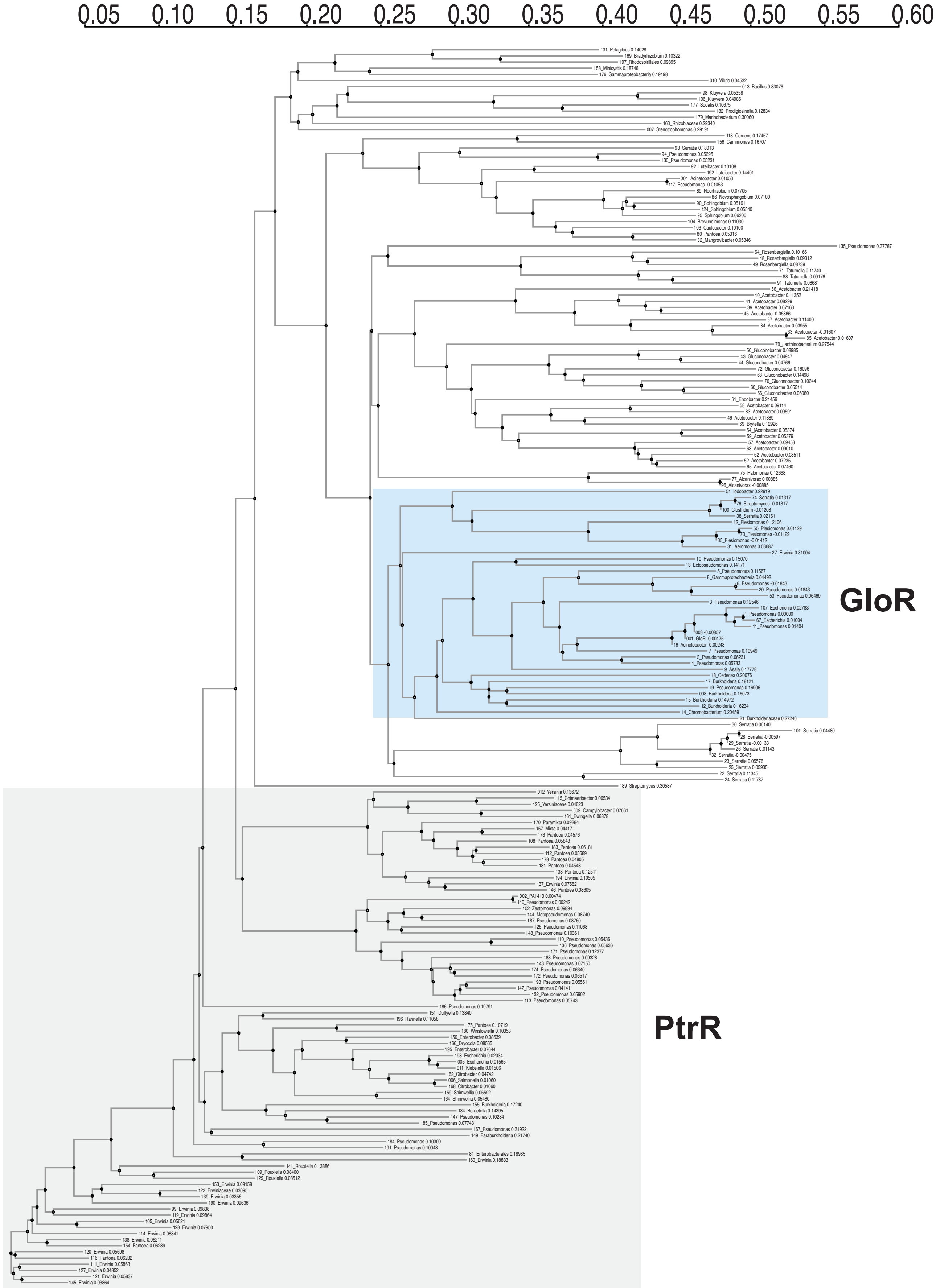

Figure S6

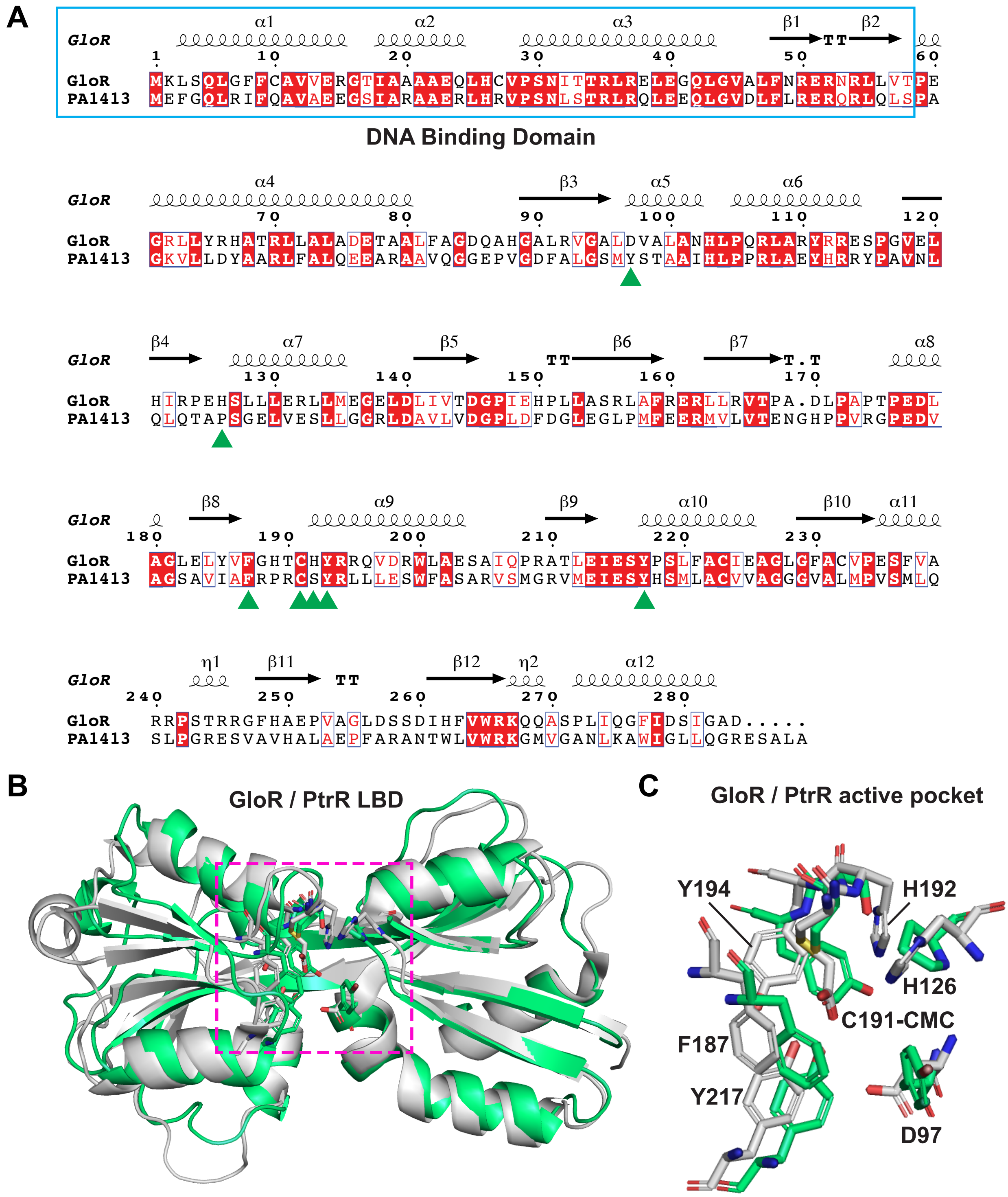

A
GloR
α1 α2 α3 β1 TT β2
GloR MKLSQLGFFCAVVERGTIAAAAEQLHCVPSNITTRLRELEGQLGVALFNRERNRLLVTPE
PA1413 MEFGQLRIFQAVAEEGSIARAAERLHRVPSNLSTRLRQLEEQLGVDLFLRERQRLQLSPA
DNA Binding Domain
α4 β3 α5 α6
GloR GRLLYRHATRLLALADETAALFAGDQAHGALRVGALDVALANHLPQRLARYRRESPGVEL
PA1413 GKVLLDYAARLFALQEEARAAVQGGEPVGDFALGSMYSTAAIHLPPRLAEYHRRYPAVNL
β4 α7 β5 TT β6 β7 T.T α8
GloR HIRPEHSLLLERLLMEGELDLIVTDGPIEHPLLASRLAFRERLLRVTPA.DLPAPTPEDL
PA1413 QLQTAPSGELVESLLGGRLDAVLVDGPLDFDGLEGLPMFEERMVLVTENGHPPVRGPEDV
β8 α9 β9 α10 β10 α11
GloR AGLELYVFGHTCHYRRQVDRWLAESAIQPRATLEIESYPSLFACIEAGLGFACVPESFVA
PA1413 AGSAVIAFRPRCSYRLLLESWFASARVSMGRVMEIESYHSMLACVVAGGGVALMPVSMLQ
η1 β11 TT β12 η2 α12
GloR RRPSTRRGFHAEPVAGLDSSDIHFVWRKQQASPLIQGFIDSIGAD.....
PA1413 SLPGRESVAVHALAEPFARANTWLVWRKGMVGANLKAWIGLLQGRESALA
B
GloR / PtrR LBD
C
GloR / PtrR active pocket
Y194
H192
H126
C191-CMC
F187
Y217
D97

Table S1. Significantly differentially regulated WT vs. Δ*gloR* transcripts

**Δ*gloR* 2 mM GO vs WT 2 mM GO (15-minute incubation)**			
**Gene**	**Log_2 Fold Change**	**Padj**	**Annotation / Prediction**
tauD1	1.687	3.93E-05	Taurine dioxygenase
PA2203	1.659	7.11E-24	Possible arginine/ornithine transporter
ssuC	1.430	2.64E-06	Aliphatic sulfonate permease
ssuD	1.405	0.01540846	Alkanesulfonate monooxygenase
PA4192	1.327	1.58E-08	Polar amino acid transport (ArtP)
PA3445	1.320	0.00012566	Hypothetical
toxR	1.122	1.93E-22	Transcriptional regulator
PA4191	1.111	6.77E-10	Isopenicillin-N synthase/ascorbate iron reductase
aguB	1.065	2.77E-31	N-carbamoylputrescine amidohydrolase
tauA	1.049	0.00181819	Periplasmic taurine-binding protein
cysW	1.037	1.26E-18	Sulfate transport
PA2088	1.027	0.00041108	Hypothetical
tonB2	1.017	0.00263937	TonB transporter
PA4195	1.012	1.93E-11	Polar amino acid transporter
PA2204	1.000	0.0101233	Possible arginine/ornithine transporter
PA2481	-1.135	4.18E-30	Possible cytochrome c
PA1412	-1.376	7.64E-11	MFS general transporter
gloR	-2.721	1.45E-117	LysR-type transcription factor
PA0712	-3.663	0	Possible toxin/antitoxin pair
PA0714	-4.681	2.24E-211	Hypothetical
PA0713	-4.749	0	Hypothetical
PA0711	-7.407	0	Possible toxin/antitoxin pair
gloA2	-7.785	0	Lactoylglutathione lyase
arqI	-7.834	0	Regulates PQS

[1]Differentially regulated genes with a log_2Fold Change greater than 2 or less than -2 are displayed.
[2]A Padj equal to zero is rounded down due to the actual value being below the displayable value.

Table S2. Data collection, phasing and refinement statistics for GloR datasets.

	Native	Glyoxal	MAD dataset		
**Data collection**					
Space group	$P\ 2_1\ 2_1\ 2$	$P\ 2_1\ 2_1\ 2$	$P\ 6_1$		
Cell dimensions					
a, *b*, *c* (Å)	99.9, 117.3, 34.3	100.5, 119.1, 34.2	149.1, 149.1, 75.8		
(°)	90, 90, 90	90, 90, 90	90, 90, 120		
			Peak	*Inflection*	*Remote*
Wavelength	1.0	1.0	0.97961	0.97944	0.91162
Resolution (Å)[a]	39.1-2.6 (2.72-2.6)	38.4-2.09 (2.15-2.09)	37.4-2.5 (2.6-2.5)	37.4-2.5 (2.6-2.5)	37.4-2.5 (2.6-2.5)
R_{merge}[b]	0.122 (1.132)	0.146 (1.284)	0.078 (1.239)	0.202 (4.063)	0.168 (3.320)
I / □*I*	8.2 (1.8)	6.2 (1.5)	16 (2.1)	17.4 (15.7)	10.1 (1.7)
Completeness (%)	99.9 (100)	98.9 (91.5)	99.5 (97.6)	99.0 (92.4)	99.4 (96.0)
Redundancy	6.9 (7.2)	9.2 (8.8)	12.9 (11.7)	17.4 (15.7)	17.5 (17.2)
**Refinement**					
Resolution (Å)	38.0-2.6 (2.7-2.6)	36.9-2.09 (2.17-2.09)	37.4-2.5 (2.56-2.50)		
No. reflections	12998 (1393)	24735 (1202)	33367 (1301)		
R_{work} / R_{free}[c]	23.3/30.0 (38.6/51.1)	21.9/24.8 (32.1/36.0)	21.8/25.7 (35.9/41.4)		
Number of subunits	2	2	4		
Number of Residues	399	395	788		
No. atoms	3120	3242	6014		
Protein	3120	3110	5973		
Ligand/ion	0	0	34		
Water	0	132	7		
B-factors	64.4	44.6	91.1		
Protein	64.4	44.6	91.0		
Ligand/ion	N/A	N/A	94.2		
Water	N/A	46.3	114.2		
Ramachandran Favored	96.2	97.4	96.1		
Ramachandran Outliers	0	0	0		
R.m.s deviations					
Bond lengths(Å)	0.008	0.009	0.009		
Bond angles (°)	1.06	1.05	1.14		
PDB Deposition ID	9NLI	9NRQ	9NLM		

[a]. Values within parentheses refer to the highest shell.

[b]. $R_{merge} = \sum\sum|I_{hkl} — I_{hkl}(j)|/\sum I_{hkl}$, where $I_{hkl}(j)$ is observed intensity and I_{hkl} is the final average value of intensity. $R_{pim} = \sum\{1/[N_{hkl}-1]\}^{1/2} \times \sum|I_{hkl}(j) — I_{hkl}|/\sum\sum I_{hkl}(j)$. $R_{meas} = \sum\{N_{hkl}/[N_{hkl}-1]\}^{1/2} \times \sum|I_{hkl}(j) — I_{hkl}|/\sum\sum I_{hkl}(j)$. $CC = \sum (x-(x))(y-(y))/ [\sum(x-(x))^2 \sum(y-(y))^2]^{1/2}$.

Supplementary Figure Legends

Figure S1. Additional EMSAs and reporter assays to assess GloR regulation. (**A**) WT MPAO1 and Δ*gloR* MPAO1 strains harboring the dual reporter system to analyze the activity of GloR regulated promoters were cultured and either not treated (NT) or treated with 2 mM GO, 4 mM GO or 4 mM MGO and reporter activity read after 3 hours incubation. Statistical analysis: *, $p < 0.05$; ****, $p < 0.0001$ by Two-way ANOVA and Šídák's multiple comparisons test. (**B**) SDS-PAGE of purified full-length GloR protein. (**C**) Size exclusion chromatography (SEC) chromatogram showing that GloR migrates as a predicted dimer. 50 µl of GloR at 100 µM (monomer) concentration was injected onto a Superose 6 increase10/300 GL column equilibrated with buffer (50 mM Tris-Cl pH 8.0, 500 mM NaCl, 1 mM TCEP). Protein was resolved at a flow rate of 0.4 mL/minute. (**D**) EMSAs of promoter regions of *arqI-gloA2*, *PA2204*, *PA4195*, and *PA1412*. Increasing amounts of purified GloR were added to a fixed amount (0.5 µM) of fluorescently labeled dsDNA probe. Un, unlabeled probe (competitive). Scr, scrambled *glo box* binding site sequence to prevent GloR from binding (non-competitive).

Figure S2. Purification and structure insights into the $GloR_{LBD}$. (**A**) SDS-PAGE gel of WT $GloR_{LBD}$ and the $GloR_{LBD}$ C191S mutant purified protein. (**B**) SEC analyses of $GloR_{LBD}$ with GO (red spectrum) and without GO (blue spectrum) using a HiLoad 16/600 column. Standards are shown in grey (dotted line) with the peaks labeled in Daltons. (**C**) Cartoon representation of $GloR_{LBD}$ tetramer. The four monomer subunits are shown as magenta, cyan, green, and yellow. (**D**) 180° rotation of the $GloR_{LBD}$ monomer with labeled α-helices (cyan), β-strands (magenta) and loop regions (wheat). (**E**) Omit map of subunit A of the unmodified structure and (**F**) modified structure with C191-CMC. Here C191 is labeled as C110. (**G**) Front and side views of the SeMet (wheat) and unmodified (cyan) structures superimposed showing the loop region which differs

between them (highlighted by the red arrow). (**H**) Ligand binding pocket residues from SeMet subunits A (orange) and C (wheat) showing the acetate hydrogen bonding versus (**I)** the same pocket in the C191-CMC modified structure (magenta). Dotted lines indicate hydrogen bond interactions.

Figure S3. **Mass spectrometry and structural modeling to interrogate C191 adducts.** (**A**) MS of $GloR_{LBD}$. Purified WT $GloR_{LBD}$ was subjected to either GO, MGO, GO for 10 minutes followed by DTT treatment, or no DTT addition control, and subjected to LC-MS. Calculated masses are shown above peaks. The theoretical mass for $GloR_{LBD}$ is 22,309.16 Daltons (Da), and $GloR_{LBD}$ modified with GO to form C191-CMC observed a mass shift of 56.84 Da (calculated). (**B**) Chemical formation (above) and atomic modeling (below) on C191 of GO CMC and MGO CEC adducts versus their corresponding precursory, unstable hemithioacetal PTMs. Hemithioacetal GO and MGO cysteine modification adducts were modelled into the density of the stable GO CMC adduct (carboxymethyl) cysteine at position 191 from the crystal structure, and then refined in phenix.refine (71) to generate an electron density map. The resulting model and map are shown for the stable CMC and CEC adducts, and more unstable hemithioacetal GO and MGO versions. Images were generated in Coot (72). Carbons are shown in green, the 1 Å $2F_o$-F_c map in blue, and the 3 Å F_o-F_c is shown in green (positive density) and red (negative density).

Figure S4. Western blot of GloR variant expression and representative plates for aldehyde killing assays. (**A**) Western blot showing GloR-FLAG levels of protein in knock-in *P. aeruginosa* strains. *P. aeruginosa* expressing GloR-FLAG variants from the native chromosome location were cultured in LB to mid-log phase, pelleted and run as whole cell lysates on an SDS-PAGE before membrane transfer and probing with an M2 anti-FLAG monoclonal antibody. GloR-FLAG

is indicated by arrows and the C191 variants labeled in brown. (**B**) Photos of plates from aldehyde killing assays where 1:10 dilutions of *P. aeruginosa* were spotted to determine CFUs. These plates are representative of 3 biological repeats.

Figure S5. Phylogenetic tree of GloR related sequences. The GloR sequence from *P. aeruginosa* PAO1 strain was used in a NCBI Blast search to identify homologs. Sequences were uploaded into Clustal Omega (33) to generate an alignment, which was curated for sequences that harbored a C191 equivalent residue before a phylogenetic tree was finally generated (also using Clustal Omega). GloR and PtrR homologs are colored blue and grey, respectively, based on their amino acid adjacent to the conserved C191 (Histidine for GloR and Serine or other residues for PtrR). Branch lengths indicate divergence, with shorter branches signifying greater similarity, typically representing substitutions per site (*e.g.*, 0.1 = 10% difference). Numbers on branches represent distance, with lower scores indicating closer relationships. Nodes (junctions) represent common ancestors. Groups of sequences branching from the same node are more closely related. Branch ends are labeled with the corresponding number to Dataset S2, the distance number and the genus of the species. Full species names and NCBI numbers which are unique to the sequences are given in Dataset S2.

Figure S6. GloR and PtrR primary and tertiary alignments. (**A**) *P. aeruginosa* GloR and PtrR alignment. The GloR sequence from *P. aeruginosa* PAO1 (PA0708) was aligned to the PtrR homolog from PAO1 (PA1413) using Clustal Omega and alignment generated using ESPript version 3 (73). GloR secondary structure information based on the deposited crystal structure (PDB code 9NLI) is given above the alignment. Key pocket residues mentioned in the text are indicated with the green arrows. A blue box highlights the DNA binding domain. (**B**) Superimposition of $GloR_{LBD}$ modified structure (grey) and a $PrtR_{LBD}$ model generated from the

P. aeruginosa sequence (locus number PA1413) with AlphaFold (green) (35). The pink dotted line box indicates the ligand binding pocket(s). (**C**) Closeup of ligand binding pocket(s) from the $GloR_{LBD}$/$PtrR_{LBD}$ overlay in *B*. CMC, carboxymethyl cysteine.
